# Glass transition of aqueous solutions involving annealing-induced ice recrystallization resolves liquid-liquid transition puzzle of water

**DOI:** 10.1038/srep15714

**Published:** 2015-10-27

**Authors:** Li-Shan Zhao, Ze-Xian Cao, Qiang Wang

**Affiliations:** 1 Beijing National Laboratory for Condensed Matter Physics, Institute of Physics, Chinese Academy of Sciences, Beijing 100190, China; 2 Department of Physics, University of Science and Technology Beijing, Beijing 100083, China

## Abstract

Liquid-liquid transition of water is an important concept in condensed-matter physics. Recently, it was claimed to have been confirmed in aqueous solutions based on annealing-induced upshift of glass-liquid transition temperature, 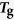. Here we report a universal water-content, 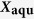, dependence of 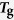 for aqueous solutions. Solutions with 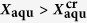 vitrify/devitrify at a constant temperature, 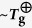, referring to freeze-concentrated phase with 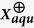 left behind ice crystallization. Those solutions with 
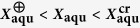
 totally vitrify at 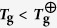 under conventional cooling/heating process though, 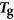 of the samples annealed at temperatures  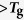 to effectively evoke ice recrystallization is stabilized at 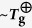. Experiments on aqueous glycerol and 1,2,4-butanetriol solutions in literature were repeated, and the same samples subject to other annealing treatments equally reproduce the result. The upshift of 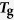 by annealing is attributable to freeze-concentrated phase of solutions instead of ‘liquid II phase of water’. Our work also provides a reliable method to determine hydration formula and to scrutinize solute-solvent interaction in solution.

The first-order liquid-liquid transition (LLT), a hot topic of condensed matter physics and materials science per se, has drawn much attention particularly from researchers in water science since 1992^1^. Simulations suggested that supercooled water, depending on temperature and pressure, can exist in two distinct forms, namely, the low-density liquid water and the high-density liquid water^2^,^3^,^4^,^5^. The first-order phase transition between the low-density and high-density phases of water is speculated to terminate at a liquid-liquid critical point (LLCP) on the phase diagram^1^,^6^,^7^,^8^,^9^,^10^,^11^. Despite vehement debate^12^,^13^, the LLCP hypothesis has since been taken as one main mechanism responsible for various anomalous properties of supercooled water^8^,^9^,^13^,^14^,^15^. Regretfully, LLCP is believed to lie at a temperature lower than the homogeneous nucleation temperature of ice (235 K), which is thus inaccessible for supercooled bulk water^16^. Consequently, it is very difficult to verify experimentally the existence of the speculated liquid-liquid transition or to investigate the properties of the so-called second critical point for supercooled bulk water. In order to circumvent this obstacle, water in aqueous solutions^17^,^18^,^19^ or confined within nanometer-sized pores^8^,^20^,^21^ comes into scope. In these two cases, ice nucleation can be effectively suppressed, and water therein can be easily supercooled down to temperatures even far below the anticipated LLCP.

Recently, it was reported that annealing-induced LLT has been observed in supercooled aqueous solutions of glycerol^18^ and 1,2,4-butanetriol^19^. Those solutions in the concentrations concerned there can totally vitrify upon cooling at a moderate rate, and the corresponding glass-liquid transition, i.e., the devitrification process, begins to appear on the heating curve at a temperature 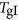. This glass-liquid transition temperature was found to shift from 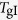 to a higher value 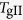 in a sample which has experienced annealing treatment, in the heating process, at a temperature 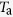 (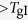) for a sufficiently long time. Besides glass transition temperature, 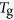, other physical properties such as the fragility, density, temperature-dependent dielectric relaxation, and hydrogen bonding state all manifest obvious difference between the precursor and the annealed samples. These annealing-induced changes in glass-transition related properties are interpreted as evidence of liquid water polymorphism in the measured aqueous solutions. The conceived liquid phases of water in the precursor and annealed solutions are termed liquid I and liquid II phase of water, respectively, in the literature. However, as also noticed by the authors of refs.^18^ and 19, such an LLT of water is always accompanied by recrystallization of ice (in the heating process at a temperature higher than the glass-liquid transition temperature). To exclude the possibility that the claimed LLT of water is per facto due to ice recrystallization-induced macroscopic phase separation, time-dependent Raman spectra were measured to trace the change in hydrogen bonding state of the sample in the course of annealing treatment. A transition from high density amorphous water-like profile to low density amorphous water-like profile is claimed to be observed, and being consequently regarded as a circumstantial evidence of LLT.

In the current work, we report systematic measurement of the water-content dependence of glass transition temperature for various aqueous solutions in an as wide as possible range of concentration, which reveals a universal behavior that can set criteria to divide the solutions into three distinct zones. We noticed that for the medium-concentration aqueous solutions, glass-liquid transition can be complicated by ice recrystallization in the heating process depending on the thermal history, and the liquid phase II actually corresponds to the freeze-concentrated phase, i.e., the portion of the solution of an elevated but definite concentration resulting from water freezing, of the annealed solutions. We repeated the annealing treatments on the same solutions as reported in refs 18,19, and reproduced the result therein, which fits well with the data for freeze-concentrated phase which can be accessed simply upon cooling in water-rich solutions and accessed via designed annealing treatment in the medium-concentration solutions. Such a glass transition behavior related to the ‘liquid II phase of water’ can be reproduced in most electrolytic and organic solutions so long as they fall within the medium-concentration zone and a proper annealing treatment is applied. Together with the comparative study by using dielectric loss spectroscopy and Raman spectroscopy on solutions in the full concentration range available, it is concluded that the ‘liquid II phase of water’ refers to the freeze-concentrated phase prepared by annealing, and the claimed LLT for water in aqueous solutions based on glass transition temperature shift seems untenable. The universal water-content dependence of glass transition temperature is of essential importance in its own right.

## Results

### Universal water-content dependence of *T*_g_

**Figure 1:**
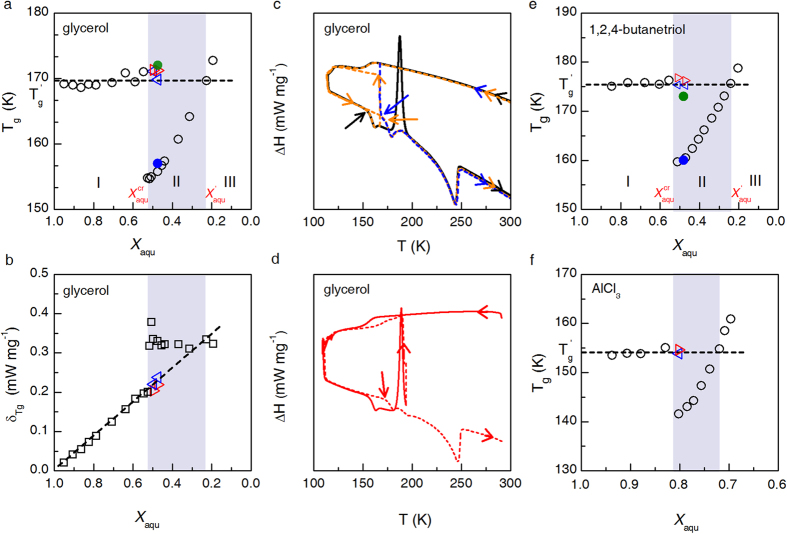
Glass transition of aqueous solutions complicated by annealing-induced ice recrystallization. (**a**) Glass transition temperature, 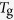, against the mass fraction of water, 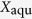, for glycerol solutions (empty circle) obtained under conventional cooling/heating process. In those
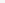
solutions with a 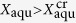, ice precipitation occurs first in cooling process. The freeze-concentrated phases vitrify at a constant 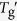, and the corresponding 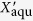 can be directly read from the monotonous part of the 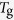 vs. 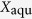 curve at the point where 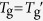. Here 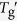 = 170 K, 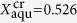, 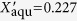. For comparison, 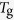 data for the liquid I (blue solid circle) and liquid II (olive solid circle) phases of water in glycerol solution with 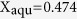 reported in ref. 18 are presented. (**b**) Solution mass-normalized heat flow change at glass transition, 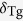, as a function of 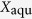 for glycerol solutions. The blue and red hollow triangles in (**a,b**) denote the data points for freeze-concentrated phases of glycerol solutions with 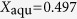 and 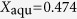 subjected to temperature protocol 2 and protocol 4 (see below), respectively. (**c**) DSC thermograms for three distinct temperature protocols demonstrated with the glycerol solution of 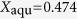. Protocol 1 (black line): Conventional cooling/heating process; protocol 2 (blue line): The cooling process is terminated at 167 K and maintained there for 230 min., followed by heating to room temperature; protocol 3 (orange line): The heating process is interrupted at 167 K and maintained there for 230 min. After that the sample was again cooled down to 113 K and then heated to room temperature. (**d**) Protocol 4: The heating process is interrupted at 194 K. After maintained there for 1 min. to complete ice recrystallization, the sample was again cooled down to 113 K and then heated to room temperature. Arrows pointing at the curves indicate where 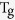 is extracted. (**e,f**) 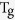 versus 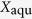
for aqueous solutions of 1,2,4-butanetriol and AlCl_3_, respectively. For the former,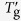 = 175 K, 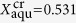, 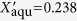; for the latter, 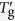 = 154 K, 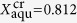, 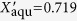. Again, the blue (protocol 2) and red (protocol 4) triangles corresponding to measurements with annealing treatment, and data from ref. 19 (solid circles), are presented.

The water-content dependence of 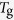 was measured for the aqueous solutions of glycerol, 1,2,4-butanetriol and AlCl_3_, which manifests a quite universal feature (Fig. 1) when a conventional one-round cooling/heating process at a moderate rate ( ≤ 20 K/min.) was adopted (for the typical DSC thermograms of solutions with different water contents, see Supplementary Fig. 1). The universal water-content dependence of 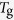 can also be verified in other solutions of electrolytes and organic substances, and their mixtures (see Supplementary Fig. 2. Measurements on more than 20 different solutions will be published elsewhere). Remarkably, for a given solute, the solutions of variable water content, or correspondingly concentration, can be divided into three distinct zones following the behavior of glass transition.

In Fig. 1a, 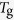 for the aqueous glycerol solutions, with increasing water content, first drops monotonically. The corresponding solutions with a mass fraction of water, 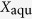, below a critical value, i.e., 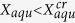, can totally vitrify in the cooling process (see Supplementary Fig. 1b,c). This monotonous drop of 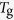 value with increasing water content results from the plasticizer effect of water. In contrast, in those solutions with 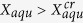, precipitation of primary ice occurs at first in the cooling process (a pronounced endothermal peak appears prior to the glass transition on DSC curve, see Supplementary Fig. 1a), thus glass-liquid transition temperatures stabilized at a constant value, 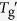, were measured, which arises from the devitrification of the freeze-concentrated phase. The water content for the freeze-concentrated phase,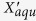, which corresponds to the hydration formula for the given solute, can be directly read from the monotonous part of the 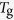 vs. 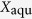 curve at the point where 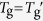. With 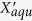 and 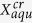 at hand, the aqueous solutions can be divided into three distinct zones, marked as I, II, III in Fig. 1a, with regard to the glass transition behavior. For aqueous glycerol solution, 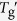 = ~170 K, 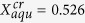, and 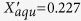. Since the freeze-concentrated phase of the water-rich solutions has a nearly constant concentration, it leads to a linear relationship between 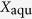, or the amount of freeze-concentrated phase, and the heat flow change per unit mass of solution at 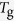, denoted as 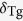 (Fig. 1b). Reasonably, the linear dependence of 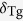 upon 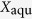 can extend into zone II so long as ice precipitation has been successfully evoked prior to glass transition via a proper cooling/heating procedure, as demonstrated by experiment on glycerol solutions with 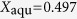 and 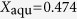 subjected to cooling/heating procedures specified in protocol 2 (Fig. 1c) and protocol 4 (Fig. 1d). This feature confirms the picture that after ice precipitation, only the freeze-concentrated phase is involved in the vitrification and devitrification processes for solutions with some excessive water, i.e., 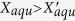.

For those solutions falling in zone II with 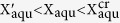, which do contain more water than required for the hydration of the solute molecules or ions, recrystallization of excessive water may be suppressed or not depending on the cooling/heating procedure applied. In this case, glass transition can be complicated by annealing-induced recrystallization of water (Fig. 1a,e), that annealing treatment at a temperature 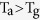 that effectively evokes ice recrystallization will result in measuring an upshifted 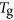 which corresponds to 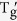 for the freeze-concentrated phase. This seems to be the culprit for the misconceived liquid-liquid transition of water in previous researches.

### Annealing-induced ice recrystallization and LLT

In order to verify our judgment, measurement on the aqueous glycerol solution investigated in ref. 18 will be exactly reproduced, and the sample will also be subjected to particularly designed annealing treatment. For the sample concerned, it has a molar fraction of glycerol of 0.178 which corresponds to 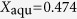. It simply vitrifies completely upon cooling at a moderate rate of 20 K min^−1^. In the subsequent heating process, the devitrification process starting at 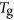 = 155.8 K is followed by ice recrystallization as revealed by a pronounced exothermal peak on the DSC heating curve with an onset temperature 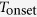
~180 K (black line in Fig. 1c). However, we call for attention to the fact that such ice recrystallization can be deliberately manipulated by designing proper cooling/heating processes, namely, (1) the cooling process is terminated at a temperature 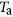 (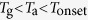), and maintained there for a sufficiently long time, e.g., for 230 min. at 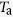 = 167 K, which is then followed by heating up to room temperature (protocol 2, blue dashed line in Fig. 1c). In the annealing process, both ice recrystallization and liquid-glass transition have finished; (2) the heating process starting from 113 K is interrupted at a temperature 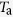 (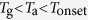) and maintained for a sufficiently long time, e.g., for 230 min. at 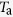 = 167 K. After that, the sample was again cooled down to 113 K and then heated up to room temperature (protocol 3, orange dashed line in Fig. 1c). One sees that the glass-liquid transition temperature thus measured in the heating process shifts to a higher value, in fact corresponding to 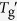 for the freeze-concentrated phase. In Fig. 1d, the sample was immediately cooled down to 113 K from 194 K, obviously ice recrystallization has been brought to the end, thus also a transition temperature 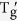 was subsequently measured. It is noteworthy that even annealing at such a high temperature (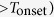 for sufficiently long time in the cooling process, ice recrystallization can be equally effectively evoked (see Supplementary Fig. 3).

With these different annealing treatments, the exothermal peak corresponding to ice recrystallization are absent on the DSC heating curve. However, the endothermal peak corresponding to the melting process of recrystallized ice at ~245 K always makes its presence. Moreover, the position and area of this endothermal peak remain unchanged irrespective of the different thermal histories, as it corresponds to a complete ice recrystallization in the sample. Figure 1a,c clearly show that the residual glycerol solutions left behind ice recrystallization vitrify totally at ~170 K that corresponds to 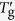 of aqueous glycerol solutions (Fig. 1a). The low-temperature phase of the annealed aqueous glycerol solutions within Zone II, similar to those in zone I with 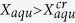, comprises a mixture of crystallized ice and vitrified freeze-concentrated phase of the same water content specified by 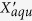, which corresponds to the hydration formula of glycerol·1.5 H_2_O. Without contribution from the precipitated ice the 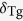 data points of the annealed samples with 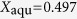 and 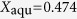 do fall on the linear extension of 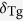 data for solutions in zone I (Fig. 1b).

We want here to emphasize that the values of 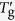 ~ 170 K for the freeze-concentrated phases formed through various annealing treatments (Fig. 1c,d) agree well with that for the suggested ‘liquid II phase of water’ in glycerol solution with 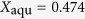
18. Therefore, conclusion can be drawn that the observed change in glass-liquid transition temperature of supercooled glycerol solutions there arises from macroscopic phase separation evoked by annealing treatment, rather than from the claimed liquid-liquid phase transition.

This kind of annealing-induced ice recrystallization and macroscopic phase separation can be observed in aqueous solutions of other electrolytes and organic substances so long as the solution falls within the respective Zone II. Figure 1e,f show the corresponding results for aqueous solutions of 1,2,4-butanetriol and AlCl_3_, 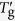 = 175 K, 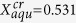, and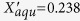 for 1,2,4-butanetriol, while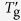 = 154 K, 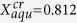, and 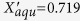 for AlCl_3_. In Fig. 1e, the two data points that are used to justify the existence of ‘liquid II phase of water’ in ref. 19 are present, again the claimed glass transition temperature for the liquid II phase is roughly 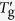 for freeze-concentrated phase. More examples are presented in Supplementary Fig. 2. For effectively inducing complete ice recrystallization, annealing temperature and time should be chosen with regard to the solute type and solution concentration. As seen from Supplementary Fig. 3a,b, for a lower 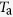 (relative to 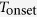 which rises with increasing concentration) a longer annealing time will be needed. For example, ice recrystallization in glycerol solution with 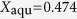 can be achieved by annealing for 230 min. at 167 K, however, at 164 K this is not done even for 480 min. The degree of ice recrystallization and consequently the concentration of freeze-concentrated phase can be evaluated by the exact value of 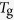 of freeze-concentrated solution and the area of the exothermal recrystallization peak (see Supplementary Fig. 3c). An incomplete ice recrystallization may imply a lowered 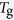 for the freeze-concentrated phase. By the way, ice recrystallization can also be evoked in the cooling process at a temperature 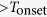, but generally speaking, it can be more easily evoked on the heating process because the previous vitrification/devitrification process can strongly promote ice recrystallization (see Supplementary Fig. 3d).

### Dielectric loss spectrometric measurement

**Figure 2:**
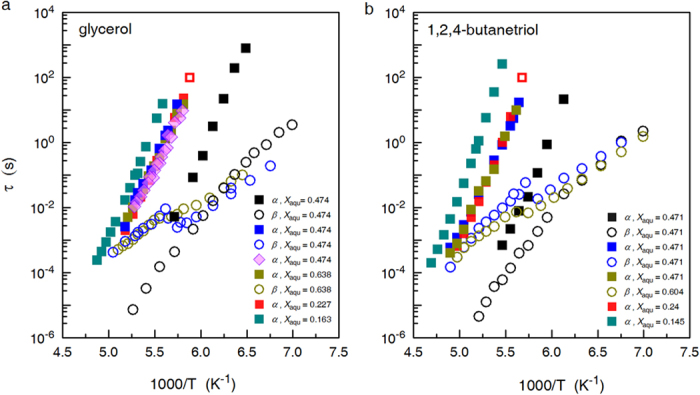
Temperature-dependent relaxation times for aqueous solutions of glycerol and 1,2,4-butanetriol subject to different thermal histories. (**a**) The relaxation times 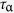 and 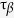 for aqueous glycerol solutions with 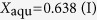, 0.474(II), 0.227, and 0.163(III), respectively. Black symbols: the sample with 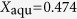 was cooled from room temperature down to each targeted temperature. After each measurement, the sample was then heated up to room temperature so as to avoid ice recrystallization and to ensure that the measured spectra correspond to a single supercooled liquid solution; Blue symbols: the cooling process was interrupted at 183 K where the sample was annealed for 150 min. After that, the sample was cooled down to 140 K, and then heated up to room temperature accompanied by spectral measurement; Magenta symbols: 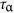 for glycerol solution with 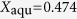 reported as ‘liquid II phase of water’ in ref. 18; Dark yellow, red, and dark cyan symbols: the samples were heated from 140 K up to room temperature accompanied by spectral measurement; Red hollow square: 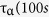) for glycerol solution with 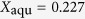. (**b**) Corresponding results for aqueous 1,2,4-butanetriol solutions. Blue symbols: the cooling process was interrupted at 195 K and maintained for 120 min. to ensure a complete ice recrystallization. After cooling down to 140 K, the sample was heating up to room temperature accompanied by spectral measurement. Other colored symbols denote the data points obtained following correspondingly the same procedure as in (**a**). For the two high-concentration samples in both cases, the 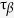 values are unavailable from fitting (see Supplementary Fig. 4).

Besides glass transition temperature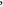 the dielectric relaxation time versus temperature relationship for aqueous solutions of different concentrations subject to annealing treatment or not can also be applied to resolve the puzzle. Dielectric loss spectra are fitted according to Havriliak-Negami relaxation functions (for more details see Supplementary Fig. 4). The relaxation times 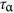 and 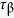, referring to the *α* process and the Johari-Goldstein (or slow *β*) process^22^, respectively, are plotted in Fig. 2 as a function of 1000/T. For both glycerol and 1,2,4-butanetriol solutions, samples falling in all the three zones and that with 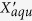 are investigated, with the samples in zone (II) also subject to annealing treatment. Data from ref. 18 are included for comparison.

Obviously, the 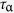 versus 1000/T relationships are very similar for glycerol solutions with 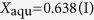, 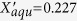, and for the annealed sample with 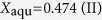. 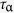 has been widely regarded to be related to the primary structural relaxation and glass transition^23^,^24^. The temperature where 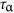 ~100 s is very close to 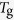 determined on the basis of DSC measurement (see red hollow square in Fig. 2a). Therefore, it is reasonable to suggest that the 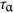 versus 1000/T relationship mainly reflects the structural relaxation of freeze-concentrated phase. That the concentrations of the freeze-concentrated phases in sample of zone I and in annealed sample of zone II are very close to 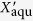 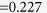
results in a very similar temperature**-**dependence of 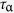. It comes as no surprise that the glycerol solution with 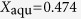
as claimed containing ‘liquid II phase of water’, displays a similar 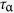 versus 1000/T curve. Figure 2b tells the same story on the 1,2,4-butanetriol solutions, where the sample with 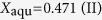 was cooled down to 195 K, maintained there for 120 min. to ensure a complete ice recrystallization. Again, the existence of ‘liquid II phase of water’ seems to be mistakenly speculated basing on the behavior of the aqueous solutions falling in zone II.

### Raman spectrometric measurement

**Figure 3:**
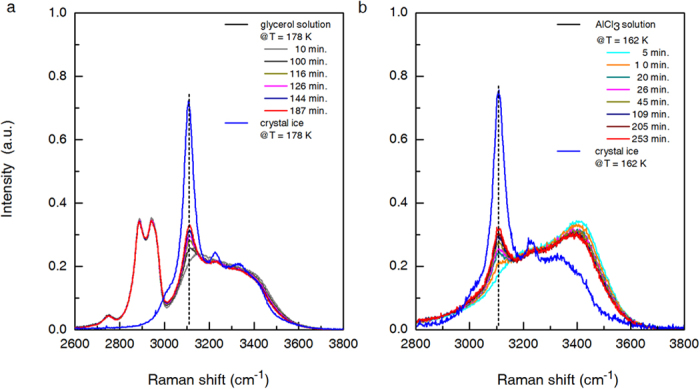
Time-dependent Raman spectra of aqueous solutions in zone II of glycerol and AlCl_3_ under annealing treatment. (**a**) Raman spectra for aqueous glycerol solution with 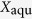 = 0.474 maintained at 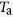 = 178 K for a time interval up to 187 min. (**b**) The corresponding results for aqueous AlCl_3_ solution with 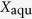 = 0.812 annealed at 162 K for a time interval up to 253 min. For comparison, Raman spectra of ice Ih measured at the annealing temperatures are also presented.

The claimed LLT for water in aqueous solutions established on the measurement of glass transition temperature may probably arise from ice crystallization-induced phase separation, a point of view that has drawn attention from many researchers^25^,^26^. Authors of refs 18,19 also noticed the presence of ice in their annealed samples though, but they didn’t think this is the reason for observed 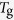 shift. To exclude this possibility, time-dependent Raman spectra were also measured during annealing treatment^18^, and the shift from the high density liquid-like profile to the low density liquid-like profile, in the sample with a molar fraction of solute of 0.178 (i.e., 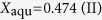 ) after annealing for 45 min.@171 K, was interpreted as a key evidence to the existence of LLT prior to ice recrystallization. Later, Suzuki and Mishima pointed out that it is inappropriate to compare the Raman spectra recorded at 171 K with those of the two kinds of amorphous ices recorded at 32 K^17^. We also traced the change in Raman spectra of glycerol solution with 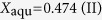 annealed at 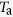 = 178 K (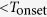) (Fig. 3a). After about 100 min., the OH-stretching band centered at ~3110 cm^−1^, which is attributable to ice Ih, becomes obviously perceivable and gains intensity with increasing annealing time. In the case of aqueous AlCl_3_ solution with 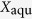 = 0.812 subject to annealing at 162 K (Fig. 3b), the OH-stretching band centered at ~3110 cm^−1^ already becomes perceivable after an annealing time of 10 min. All these observations point to the fact that for solutions falling in zone II, annealing at a temperature above glass-liquid transition temperature will effect ice recrystallization, and the subsequent measurement of a raised glass-liquid transition temperature is attributable to the freeze-concentration phase instead of a new phase of water.

In summary, by measuring the water-content dependence of glass transition temperature for various solutions in an as wide as possible concentration range, a universal feature characterized by two particular water contents, 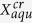, above which a constant 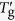 referring to the freeze-concentration phase is measured, and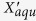 which corresponds to the freeze-concentration phase and refers to the hydration formula of the given solute, can be established. Solutions falling within zone II, i.e., with a water content 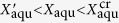, can totally vitrify at a temperature 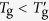 under conventional one-round cooling/heating process at moderate rate, but annealing treatments at a temperature higher than the corresponding 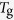 can effectively evoke ice recrystallization, resulting in the measurement of a glass-liquid transition temperature 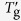 which is obviously attributable to the freeze-concentrated phase. Experiments on aqueous solutions of glycerol and 1,2,4-butanetriol that were investigated in refs. 18 and 19 were carefully reproduced, and the samples were also subject to other annealing treatments to evoke ice recrystallization so as to reproduce the raised glass-liquid transition temperature which was interpreted as evidence for the existence of ‘liquid II phase of water’. Together with dielectric relaxation and Raman spectroscopic measurements we can conclude unambiguously that the shift of glass-transition temperature by annealing treatment after vitrification/devitrification process is attributable to the freeze-concentrated phase of solution instead of the ‘liquid II phase of water’, and the glass transition temperature of the so-called ‘liquid II phase of water’ is in fact the constant 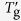 measured on all the solutions in zone **I** in which ice crystallization already occurs prior to the vitrification/devitrification. The disclosing of the universal water-content dependence of glass transition temperature is of essential importance in its own right, it provides a simple and reliable method to determine the hydration formula for solutes and to study the hydration behavior in mixture solutions.

## Methods

### Samples

High-purity water was prepared by using a Millipore Milli-Q system. The solutes, purchased from Sigma-Aldrich, are ZnCl_2_ (anhydrous, 99.99%), AlCl_3_·6 H_2_O (99%), glycerol (99.5%), ethylene glycol (anhydrous, 99.8%), sorbitol (BioUltra, ≥99.5%), and 1,2,4-butanetriol (98%).

### Differential scanning calorimetric measurement

Measurement on droplets (∼5.0 μl) of solutions was performed on a calorimeter (PE DSC8000) at a cooling/heating rate, unless specified, of 20 K min^−1^. When cooled down to 113 K, the sample would be held at this temperature for 1 min. before the heating procedure began. Glass transition temperature was extracted from the devitrification peak on the heating curve. All the DSC curves have been normalized against sample weight. Details of the particular cooling/heating protocols designed for evoking ice recrystallization in a given solution are specified in text at proper places.

### Dielectric loss spectrometric measurement

The dielectric spectra of samples, held in parallel plate sample cell BDS1308, were measured on a dielectric spectrometer (Novocontrol GmbH, Germany) equipped with a liquid nitrogen cooling system. The frequency ranges from 0.001 Hz to 10 MHz. For solutions falling within Zone II, each spectrum was measured when cooling the sample from room temperature down to the targeted temperature. After that, the sample was then heated up to room temperature so as to avoid ice recrystallization and to ensure that the measured spectra correspond to a single supercooled liquid solution. For solutions within Zone I and III and the annealed solutions within Zone II, temperature-dependent dielectric spectra were measured during heating the sample from 140 K up to room temperature.

### Raman spectrometric measurement

Temperature-dependent Raman spectra were measured by using a Jobin-Yvon HR800 Raman system. For excitation, the spectral line at 532 nm of a laser was used. The temperature was adjusted by using a cooling unit (Linkam L-600 A) and a temperature controller (Linkam TMS 94).

## Additional Information

**How to cite this article**: Zhao, L.-S. *et al.* Glass transition of aqueous solutions involving annealing-induced ice recrystallization resolves liquid-liquid transition puzzle of water. *Sci. Rep.* **5**, 15714; doi: 10.1038/srep15714 (2015).

## Supplementary Material

Supplementary Information
