# Supplementary material for: Glass transition of aqueous solutions involving annealing-induced ice recrystallization resolves liquid-liquid transition puzzle of water
Source: Sci Rep. 2015 Oct 27;5:15714. doi: 10.1038/srep15714 (PMC4621610; doi:10.1038/srep15714)
Supplement: Supplementary Information [file srep15714-s1.pdf]

SUPPLEMENTARY INFORMATION

Glass transition of aqueous solutions involving annealing-induced ice recrystallization resolves liquid-liquid transition puzzle of water

Li-Shan Zhao[1,2], Ze-Xian Cao[1] & Qiang Wang[1]

[1]Beijing National Laboratory for Condensed Matter Physics, Institute of Physics, Chinese Academy of Sciences, Beijing 100190, China

[2]Department of Physics, University of Science and Technology Beijing, Beijing 100083, China

Correspondence and requests for materials should be addressed to

Q.W (qwang@iphy.ac.cn) or

Z.-X.Cao (zxcao@iphy.ac.cn)

This supplementary information includes 4 figures.

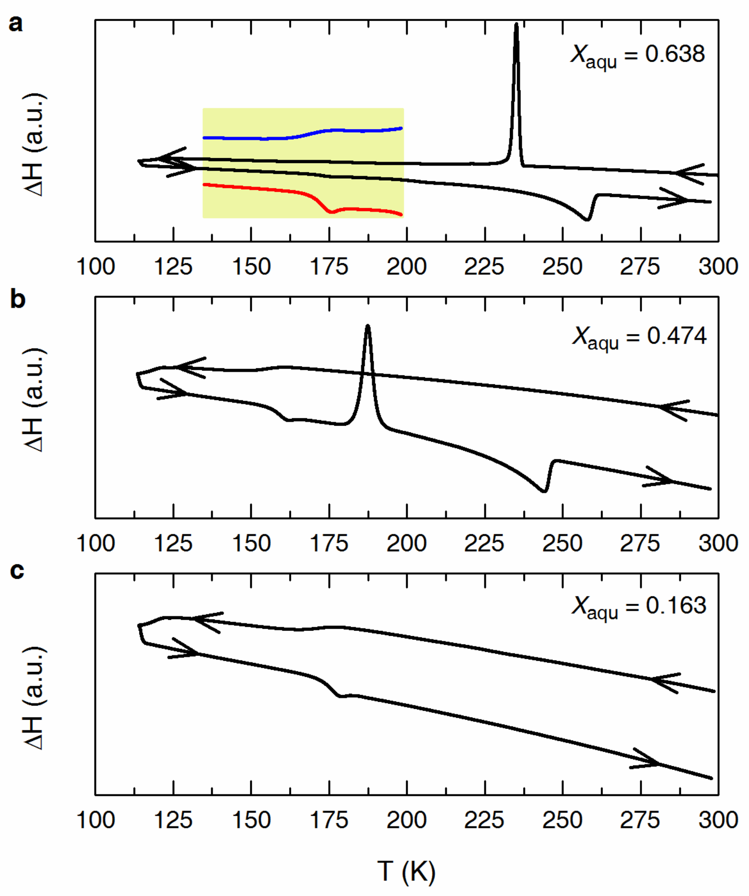

Supplementary Figure 1. DSC thermograms for aqueous glycerol solutions with a mass fraction of water of X_{aqu} = 0.638 (**a**), 0.474 (**b**), and 0.163 (**c**), falling respectively within Zones I, II, and III as specified in Fig. 1a. For water-rich solutions in zone I as illustrated with that of X_{aqu} = 0.638 (**a**), primary ice precipitates first in the cooling process as revealed by a pronounced exothermal peak. The subsequent vitrification process involves only the freeze-concentrated phase. On the heating process, devitrification occurs prior to the melting of primary ice which is revealed by an endothermal peak. For solutions in zone II as illustrated with that of X_{aqu} = 0.474 (**b**), the sample vitrifies totally in the cooling process, and devitrification in the heating process is followed by the recrystallization of ice as revealed by a pronounced exothermal peak. This is particularly the case for those solutions with a X_{aqu} apporaching $X_{\mathrm{aqu}}^{\mathrm{cr}}$. For solutions in zone III as illustrated with that of X_{aqu} = 0.163 (**c**), only vitrification and devitrification processes are observed. Arrows indicate the direction of temperature scanning. Scanning rate: 20 K min^{-1}.

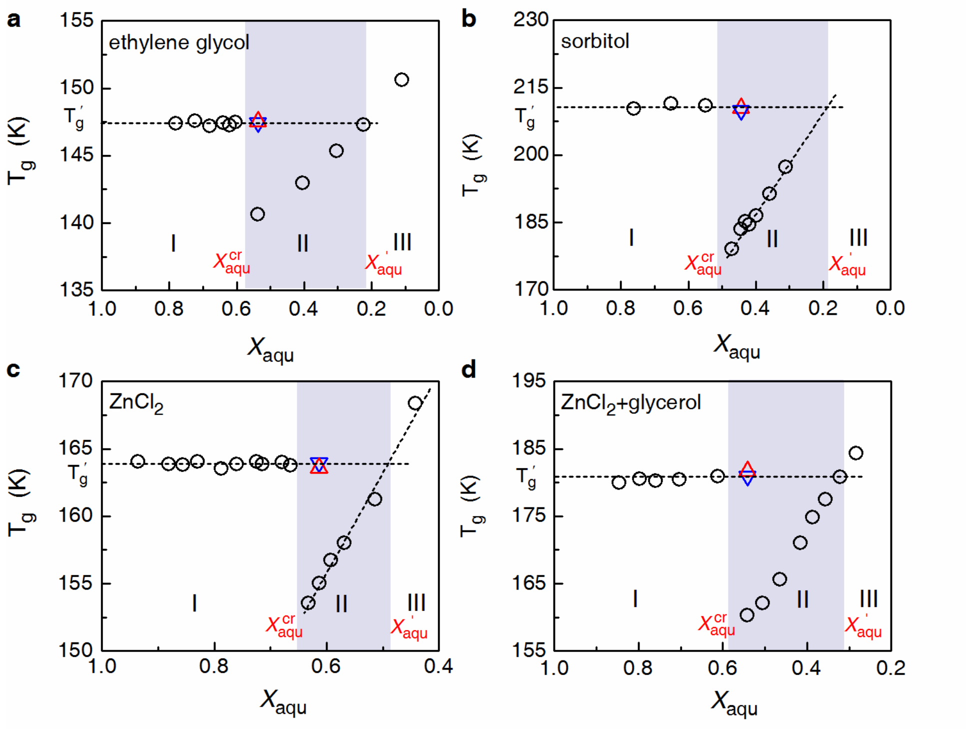

Supplementary Figure 2. Glass transition temperature versus mass fraction of water for aqueous solutions of (**a**) ethylene glycerol, (**b**) sorbitol, (**c**) $ZnCl_2$, and (**d**) $ZnCl_2$ + glycerol (molar ratio 1:1). Black circles: T_g measured in the conventional one-round cooling/heating process, i.e., protocol 1 adopted as in Supplementary Fig.1 and in Fig. 1c (black solid line); Blue and red triangles: T_g of the freeze-concentrated phase actualized by the cooling/heating process specified in protocol 3 (orange line in Fig. 1c) and protocol 4 (Fig. 1d), respectively. Here, T_g' = 147 K, X_{aqu}^{cr}= 0.575, and X'_{aqu}= 0.216 for ethylene glycerol solutions; T_g' =211 K, X_{aqu}^{cr} = 0.514, and X'_{aqu} = 0.188 for sorbitol solutions; T_g' =164 K, X_{aqu}^{cr}= 0.652, and X'_{aqu} = 0.488 for $ZnCl_2$ solutions; and T_g' =181 K, X_{aqu}^{cr} = 0.586, X'_{aqu} = 0.315 for $ZnCl_2$ + glycerol (molar ratio 1:1) solutions.

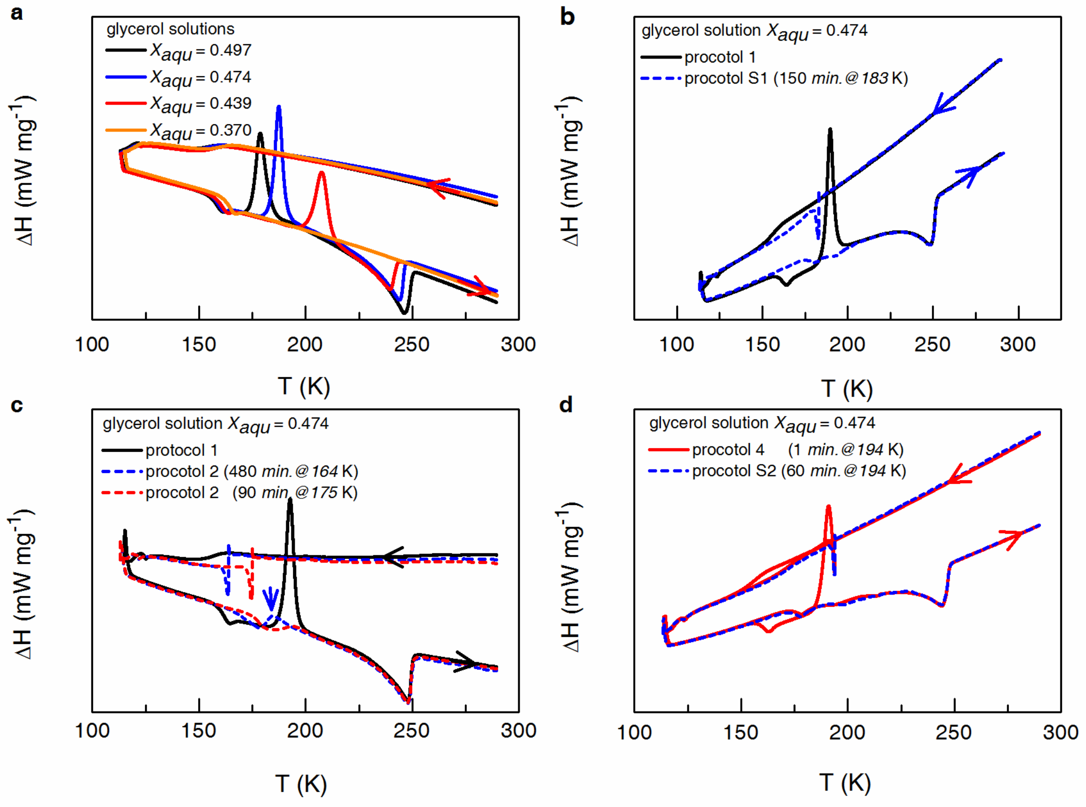

Supplementary Figure 3. Different cooling/heating protocols that effectively evoke ice recrystallization. (**a**) DSC thermograms under protocol 1 for aqueous glycerol solutions with X_{aqu} = 0.497, 0.474, 0.439 and 0.370, respectively. The onset temperature of ice recrystallization, T_{onset}, shifts steadily towards higher temperature with increasing concentrations for solutions falling within Zone II. (**b**) DSC thermograms for the aqueous glycerol solution with X_{aqu} = 0.474 under other annealing treatments. Blue line for protocol S1: the cooling process was interrupted at T_{a} = 183 K (> T_{onset} = 180 K), and maintained for 150 min. After that, the sample was cooled down to 113 K and then heated up to room temperature. (**c**) DSC thermograms for the aqueous glycerol solution with X_{aqu} = 0.474 under different annealing treatments. The degree of ice recrystallization can be evaluated by the area of the exothermal peak on the heating curve and the glass transition temperature of the freeze-concentrated phase. (**d**) DSC thermograms for aqueous glycerol solution with X_{aqu} = 0.474 under cooling/heating protocol protocol 4 and S2. Red curve (protocol 4): the heating process is interrupted at 194 K, where the recrystallization of ice has been brought to the end, and maintained there for 1 min. After that, the sample was again cooled down to 113 K

and then heated up to room temperature; Blue curve (protocol S2): the cooling process was interrupted at 194 K ($T_{\mathrm{a}} > T_{\mathrm{onset}}$) and maintained there for 60 min. After that, the sample was further cooled down to 113 K and then heated up to room temperature. Obviously, both procedures result in ice recrystallization and a shifted glass-liquid transition temperature. In other words, a vitrification/devitrification process can strongly promote ice recrystallization.

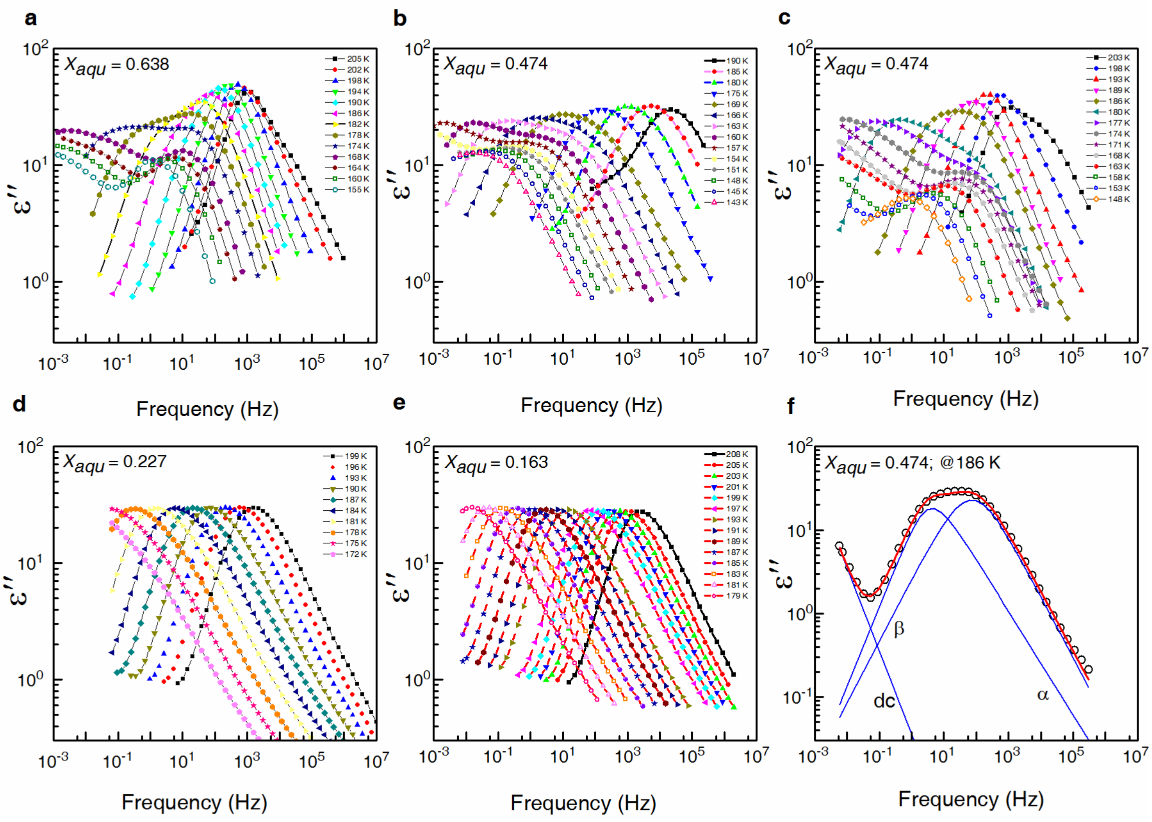

Supplementary Figure 4. Dielectric loss spectra for aqueous glycerol solutions. (**a**)-(**e**) Imaginary part of permittivity, ε'', versus temperature for aqueous glycerol solutions with an X_{aqu} of (**a**) 0.638, (**b**) and (**c**) 0.474, (**d**) 0.227, and (**e**) 0.163. With increasing temperature the main peak shifts steadily towards higher frequency. In (**a**), X_{aqu} = 0.638 > $X_{\mathrm{aqu}}^{\mathrm{cr}}$ = 0.526, spectra were measured during sample heating from 140 K up to room temperature. At 140 K, the sample comprises a mixture of crystallized ice and vitrified freeze-concentrated phase with X_{aqu} = X'_{aqu}= 0.227. For each targeted temperature point, spectral collection ran automatically when the sample temperature is within 0.3 K from the targeted temperature value. In (**b**), before measurement the sample was cooled from room temperature down to each targeted temperature point. This procedure can effectively prevent ice recrystallization, thus the measured spectra only refer to the supercooled liquid solution. In (**c**), the sample with X_{aqu} = 0.474 was first cooled down to 183 K and maintained there for 150 min., and then cooled down to 140 K. As the case for solution with X_{aqu} = 0.638, the sample with X_{aqu} = 0.474 at 140 K comprises a mixture of recrystallized ice and vitrified freeze-concentrated phase with X_{aqu} = X'_{aqu}= 0.227. The spectra were obtained during heating

process. In (**d**) and (**e**) where the solutions with X_{aqu} = 0.227 and 0.163 fall within zone III, the spectra were measured during heating process from 140 K up to room temperature. (**f**) Fitting of dielectric loss spectra with the Havriliak-Negami function, as exemplified with the data measured on the sample with X_{aqu} = 0.474 at 186 K, where 'dc' denotes the dc conductivity, 'α' and 'β' refer to the α process and the Johari-Goldstein (or slow β) process, respectively. Here τ_{α}=53,27 ms; τ_{β}= 2.29 ms when both parameters are allowed to change in the fitting process. In order to acquire a sense of the indetermninacy with the fitting parameters, the intervals for τ_{α} and τ_{β}, in which that when one parameter is fixed, a satisfactory fitting can still be made by varying the other, are given. For τ_{α}, it is 37.42~75.47 ms, while for τ_{β} this is 1.64~3.25 ms.
